# Supplementary material for: The Role of the Bone Morphogenetic Protein Antagonist Noggin in Nucleus Pulposus Intervertebral Disc Cells
Source: Int J Mol Sci. 2024 Nov 2;25(21):11803. doi: 10.3390/ijms252111803 (PMC11546912; doi:10.3390/ijms252111803)
Supplement: Supplementary file 1 [file ijms-25-11803-s001.zip › ijms-3216306-supplementary.pdf]

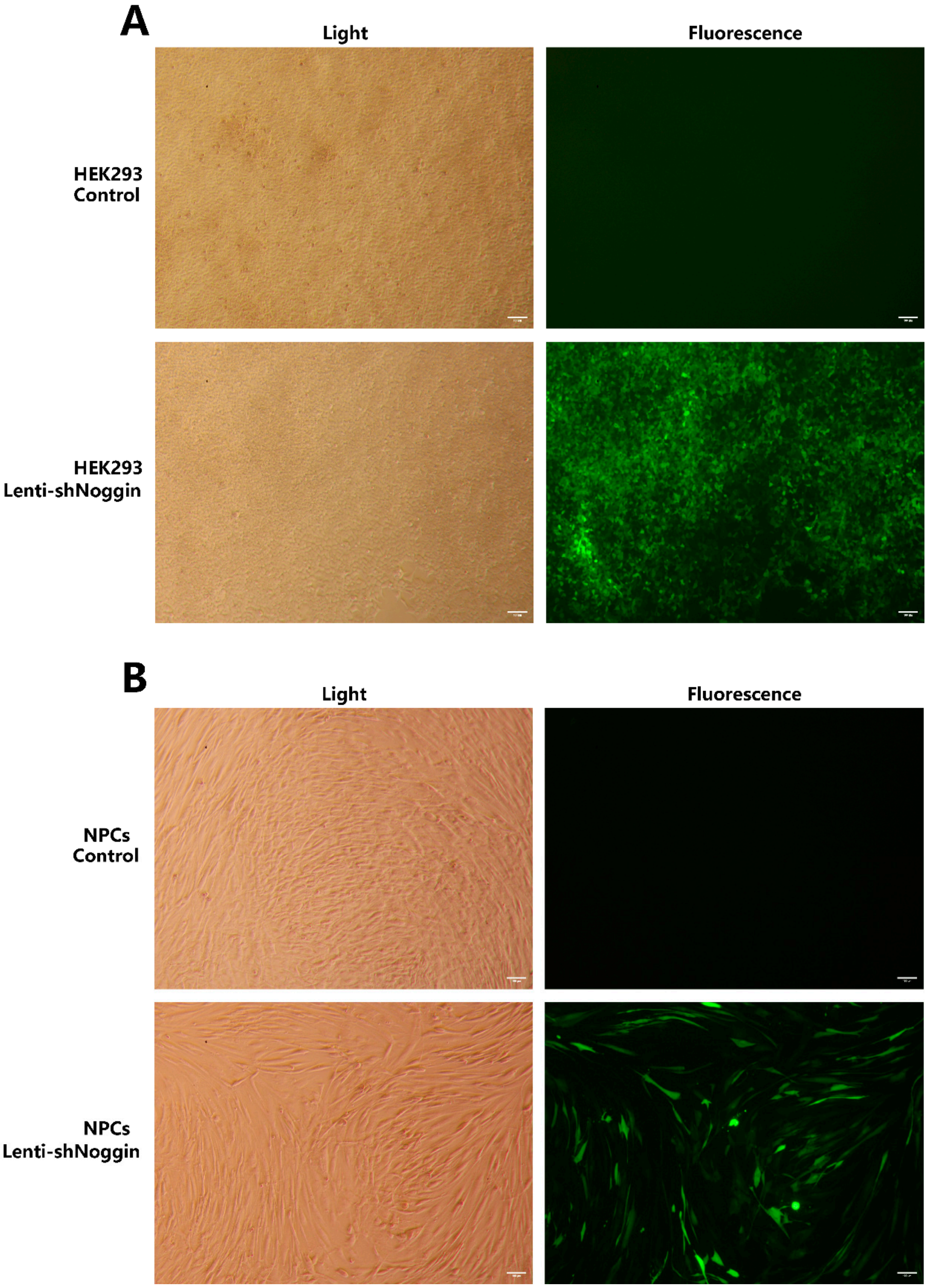

Supplementary Figure S1. Transduce **(A)** HEK293 and **(B)** NPCs with Lenti-shNoggin successfully (Scale bar: 100 μm; n=3).
